# Thin-Film MEMS Resistors with Enhanced Lifetime for Thermal Inkjet

**DOI:** 10.3390/mi11050499

**Published:** 2020-05-14

**Authors:** Elkana Bar-Levav, Moshe Witman, Moshe Einat

**Affiliations:** Department of Electrical and Electronic Engineering, Ariel University, Ariel 4070000, Israel; elkanabar@gmail.com (E.B.-L.); moshew@ariel.ac.il (M.W.)

**Keywords:** 2D printhead, 3D printing, thermal inkjet, thin-film resistors

## Abstract

In this paper, the failure mechanisms of the thermal inkjet thin-film resistors are recognized. Additionally, designs of resistors to overcome these mechanisms are suggested and tested by simulation and experiment. The resulting resistors are shown to have improved lifetimes, spanning an order of magnitude up to 2 × 10^9^ pulses. The thermal failure mechanisms were defined according to the electric field magnitude in three critical points—the resistor center, the resistor–conductor edge, and the resistor thermal “hot spots”. Lowering the thermal gradients between these points will lead to the improved lifetime of the resistors. Using MATLAB PDE simulations, various resistors shapes, with different electric field ratios in the hot spots, were designed and manufactured on an 8″ silicon wafer. A series of lifetime experiments were conducted on the resistors, and a strong relation between the shape and the lifetime of the resistor was found. These results have immediate ramifications regarding the different printing apparatuses which function with thermal inkjet technology, allowing the commercial production of larger thermal printheads with high MTBF rate. Such heads may fit fast and large 3D printers.

## 1. Introduction

One of the existing technologies for ink/fluid printing onto carrying media is the thermal inkjet printer head [1,2,3]. This head is composed of a chamber containing the print fluid. On one of the walls of the chamber there is an electrical resistor designed to heat to high temperatures [4]. In addition, there is a nozzle through which the print fluid can be ejected. There was a time where the future of inkjet technology for printing was questionable among printing experts, in light of the laser printing technique. But experience shows that the opposite has happened. Not only is the inkjet concept not vanishing, in fact, to the contrary, it is developing and penetrating new regimes suggesting amazing possibilities such as cell sorting and single-cell lysis [5,6,7], medical applications [8], fluid micromixer [9], DNA droplets [10], organic transistors [11], silver nanoparticles printing for microelectronics [12] and many more. A company called “Nano-dimension” has developed a printer that prints together conductor and insulator. This technology enables printing an electrical PCB, printed antennas and other applications that may improve electrical design. Similar technology is also used for microvalves and micropumps based on thermal bubble actuated microfluidic chips [13]. A micro-synthetic jet [14] is also based on micro heaters technology. More complicated microheaters are used for atomization of high-viscosity fluids [15]

In a typical thermal inkjet arrangement, when a current is applied to the resistor for a short time (a few microseconds) [16], it heats the surrounding fluid in its immediate vicinity and causes local boiling (micro-boiling, MB). The rapid pressure rise forces liquid at a distance from the resistor into the nozzle and out of the chamber. The rapid temperature rise and resulting temperature gradients on the resistor shorten its lifetime and therefore the lifetime of the entire head. This lifetime is shorter compared to other printer heads, such as the piezoelectric head [17].

As an example of the ramifications of this, when the ink is depleted in a thermal inkjet head the entire head is replaced, as it does not make sense to refill and continue its use. Contrarily, piezoelectric heads are refilled with ink as their lifetime is greater than a single depletion cycle. Despite this and other advantages of the piezo head [18,19], the thermal inkjet head is the more common product in many applications as it is cheaper and simpler to manufacture. 

For domestic paper printing the existing lifetime is satisfactory, but for 3D printing (additive manufacturing), where much higher operation cycles are needed for a single print, a higher lifetime is vital.

Since the central disadvantage of the thermal printhead is its shorter lifetime [20,21,22,23], the isolation of failure mechanisms and the design of more robust resistors would create a greater incentive for production and manufacturing. Park et al and Lim et al’ researches [24,25] present studies on the subject of lifetime enhancement by changing the printhead micro structure and changing the metal composition and thickness of the resistor. McGlone et al’ research [26] describes more than 10^7^ pulses obtained by the use of amorphous metal thin films. Bendong et al’ research [27] presents a concept where the heating is done with an induction element that has no physical connection to the power source. 

In this paper, two failure mechanisms of the thermal inkjet resistor are recognized. One is related to the sharp temperature gradient at the conductor–resistor contact, and the second is related to hot spots on the resistor. Both tend to cause a discontinuity in the resistor and, finally, resistor failure. Additionally, designs of resistors to overcome these mechanisms are suggested and tested by simulation and experiment. Various connecting geometries between the conductor and the resistor are tested. The resulting resistors are shown to have an order of magnitude improvement in a lifetime.

## 2. Thermal Gradients in Resistors

One form of resistor geometry is the rectangular form. It has the advantage of homogeneous current density along its surface, in addition to its geometric simplicity.

Its disadvantage; however, lies in the large temperature gradient between the conductors and itself. These gradients cause mechanical strain and eventually cracking in the connecting media, which ultimately lead to component failure. So, a clear motivation is to find a configuration that reduces the temperature gradient between the conductor and the resistor as it is a failure mechanism. 

**Figure 1:**
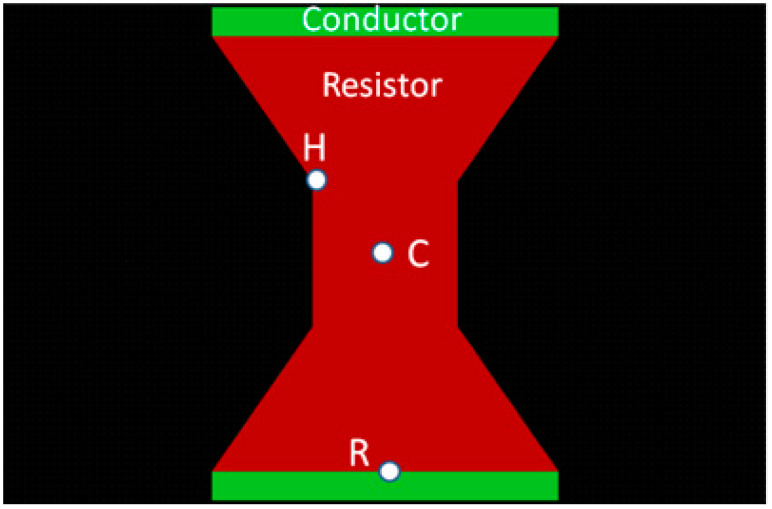
Geometry of the trapezoid resistor, with points H, C and R.

In order to reduce these gradients, a common solution is a trapezoidal or “ramp-shaped” region connecting the resistor to its conductors. Figure 1 shows the geometry of the ramp leading into the resistor and the resulting lowered temperature gradients from the conductors to the resistor. The gradient smoothing is achieved as a result of the changing resistance along the sloped trapezoidal region. Near the conductor there is lower resistance, whereas near the resistor there is higher resistance. Therefore, the sharp “jump” from the cold conductor to the hot resistor is smoothed, and the weak point of the connection now has a longer lifetime. 

**Figure 2:**
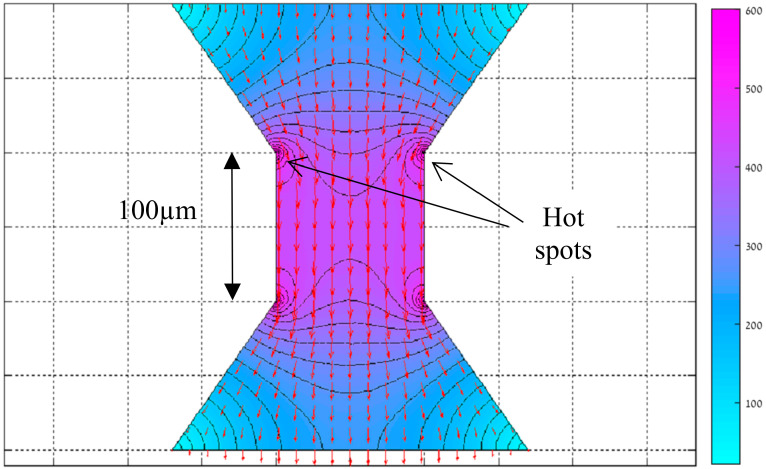
Electric field simulation of Trapezoid resistor.

However, improving one failure mechanism caused another one to rise. Despite lowering the gradients over the conductor–resistor transition region, Figure 2 shows the resulting formation of “hot spots” at the corners of the ramp–resistor interface (noted with arrows in the figure). This figure describes the results of the electric field simulation that is developed in the resistor. The simulation was carried out using a MATLAB code, written in the PDE tool. The code solved the Laplace equation in the resistor with Dirichlet boundary condition at the conductor–resistor interface, and Neumann boundary conditions everywhere else. The electric field is noted by the red arrows, together with black solid equal-potential lines. The color reflects the magnitude of the electric field. In these “hot spots” there are local maxima of the electric field and, as a result, local maxima of the current and temperature. These hot spots are extreme in both absolute temperature and in local temperature gradient, and are caused by the rise in current density in their vicinity due to the new geometry. Therefore, the hot spots are the first to be destroyed. Once there is a minor destruction and discontinuity at the hot spot point, the current must bend around it and the effect becomes worse. This leads to a rapidly-developing tear in the resistor towards the other hot spot (this is shown later at the experimental part). A full description of this failure mechanism (simulation and experiment) appears with more details in Einat et al’ research [28], as it was captured on video in a rare instance. 

In order to find the balance between these two failure mechanisms, three points of interest are defined, as seen in Figure 1; (*C*) at the Center of the resistor body; (*R*) at the center of the conductor–ramp interfaces; and (*H*) at one of the Hot spots. A certain power level and energy are required to obtain the MB effect [28] at the resistor center (*C*), but it is preferred that the temperature at (*H*), the hot spot, will not rise to much above the temperature at point (*C*). On the other hand, it is preferred that at point (*R*) the temperature will be as low as possible. Clearly these demands contradict and the goal is to find the optimal compromise that will give the longest lifetime. In order to analyze the points behavior, the electric field magnitude at points (*R*) and (*H*) is normalized to the electric field at point (*C*) as follows:

(1) $$\mathrm{En}\left(H\right)=\frac{E\left(H\right)}{E\left(C\right)},\mathrm{En}\left(R\right)=\frac{E\left(R\right)}{E\left(C\right)}$$

With E (*H*,*C*,*R*) as the absolute electric field in (V/m) at points *H*, *C* and *R,* respectively. Since the resistive layer is assumed to be uniform, the local electric field represents the local power and temperature that will develop locally in the resistor layer.

In the following section, simulations are presented which attempt to calculate these ratios, with the ultimate goal of the resulting design being the reduction of these ratios to their minimum. Ideal values are *E_n_*(*H*) = 100% (meaning that at the hot spot there is the same electric field and temperature as the resistor center) and *E_n_*(*R*) = 0% (meaning that the conductor is not heated at all). These ideal values actually imply no hotspots and no temperature gradient in the conductor–resistor interface. Approaching these values would improve lifetime characteristics.

As can easily be seen, there is a tradeoff between these two parameters—reducing one increases the other (for example, a square resistor will have no hot spots but will have a maximal gradient between the conductor and resistor border). Therefore, an optimum needs to be found.

## 3. Resistors Simulation

**Figure 3:**
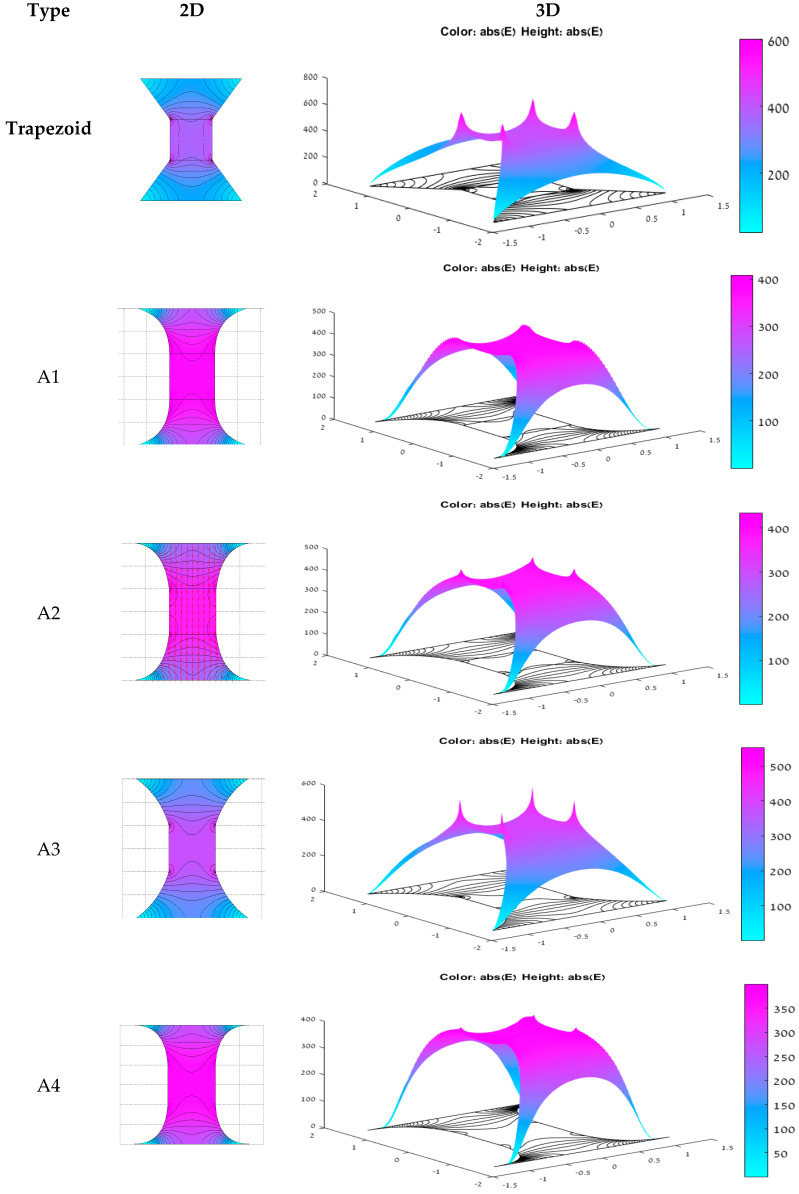
2D and 3D electric field simulation for: Trapezoid, A1, A2, A3 and A4 resistors.

In an attempt to smooth out the hot spot temperature gradients, a number of configurations were designed, simulated and experimentally tested. Several curved versions replacing the linear connection of the hot spot to the conductor in the trapezoid were tested, as seen in Figure 3. The resistor itself was kept as a 100 × 100 μm rectangular shape in all the versions for a consistent comparison, but the connection to the conductor was done through a different ramp. Figure 3 shows the different configurations, all of which have a filleted transition from ramp to resistor, labeled trapezoid, A1, A2, A3 and A4.

These fillets disperse the current density more evenly around the transition region, thereby considerably reducing the gradient in the electric field and; therefore, the temperature gradient. However, this increases the temperature gradient in the conductor–resistor interface. 

Figure 3 shows the simulation results of the electric field of each configuration. The hot spots singularity is clearly seen in the trapezoid. The reduction of the hot spots’ singularity of the other shapes can be visually compared. 

The simulation results are presented also in Table 1. It can be observed that the minimal *E_n_*(*H*) is that of A1, whereas the minimal *E_n_*(*R*) is that of the trapezoidal resistor. 

## 4. Experiment and Results

The specimens were fabricated as thin-film resistor shapes on an 8″ diameter and 500 µm thickness silicon wafer, with a 1000 µm oxidation layer. On top of the wafer, two consecutive layers were evaporated—the first of a resistive 500 nm tantalum nitride (Ta-N) with a sheet resistance of 30 Ω, and the second of a conductive 100 nm copper layer and a 500 nm gold coating layer. For good adhesion between the resistive layer and the conductive layer, a 10 nm titanium layer was also evaporated. After the evaporation of each layer, photolithography and etching processes were preformed to create the resistor’s and conductor’s shape. Each resistor body had a 100 × 100 μm rectangular shape, 30 Ω resistance, and the geometry of the different ramps is seen in Figure 3. 

**Figure 4:**
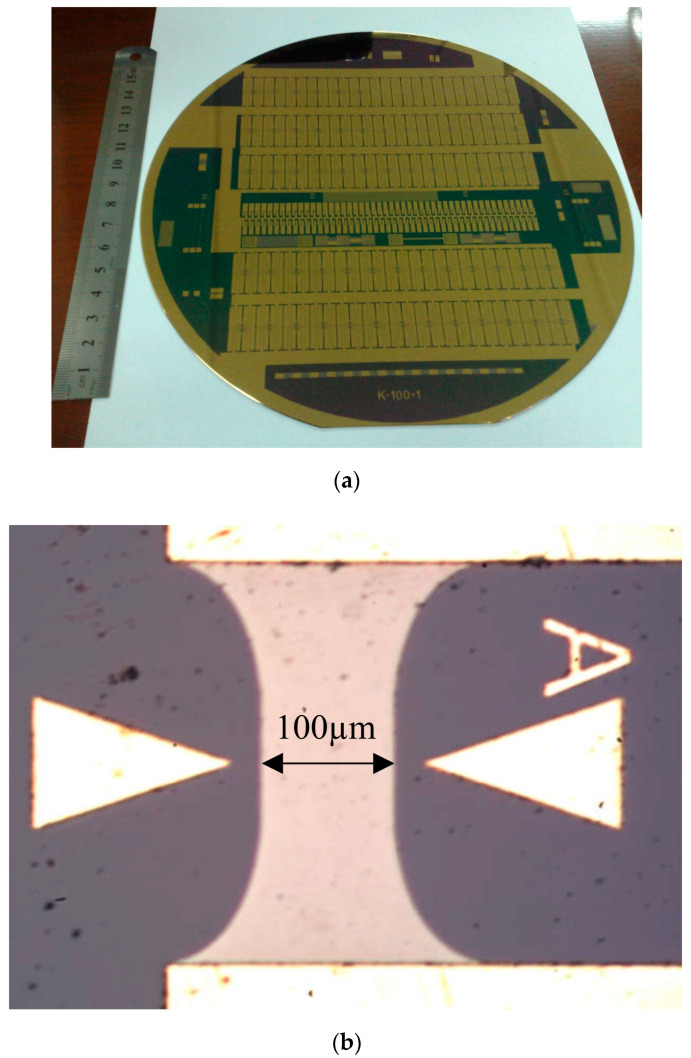
(**a**) Test 8″ Wafer, (**b**) A2 resistor.

**Figure 5:**
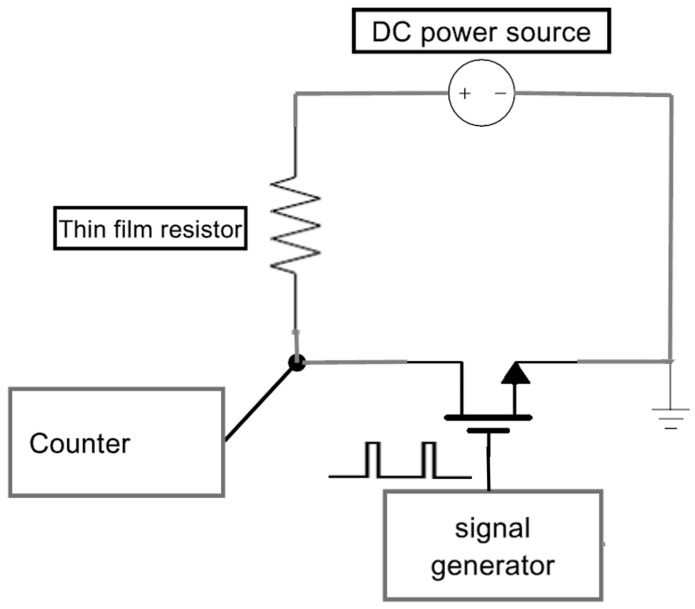
Experiment setup diagram.

The wafer and one of the planned resistors can be seen in Figure 4a,b. For the purpose of experimental testing of the simulation results, a series of experiments were conducted on the different resistors. The wafer was connected to an electric circuit depicted in Figure 5.

**Figure 6:**
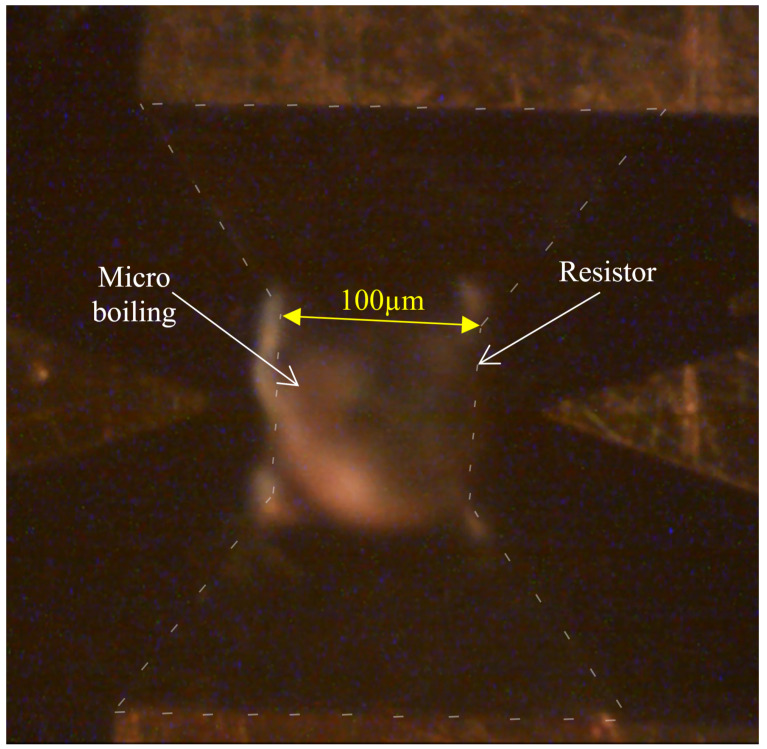
Micro-boiling process.

The experiment was conducted 5–6 times per resistor, under a constant current of 0.5 A, uniform for all resistor shapes. The circuit supplied a pulse repetition frequency (PRF) of 33 kHz from a signal generator, each pulse having a duration of 5 microseconds. It is important to note that MB had been confirmed [28] with this setup and parameters prior to experimentation, as seen in Figure 6. When a current pulse is given to the resistor, a rapid heating occurs, the fluid above the resistor heats up and a bubble grows above the resistor during the pulse duration (Figure 6). The picture was taken using a stroboscope.

**Figure 7:**
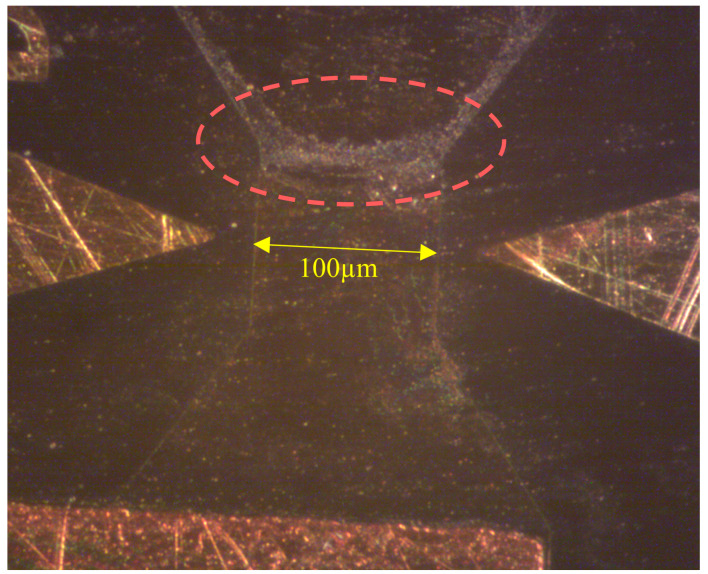
Trapezoid resistor burn out.

**Figure 8:**
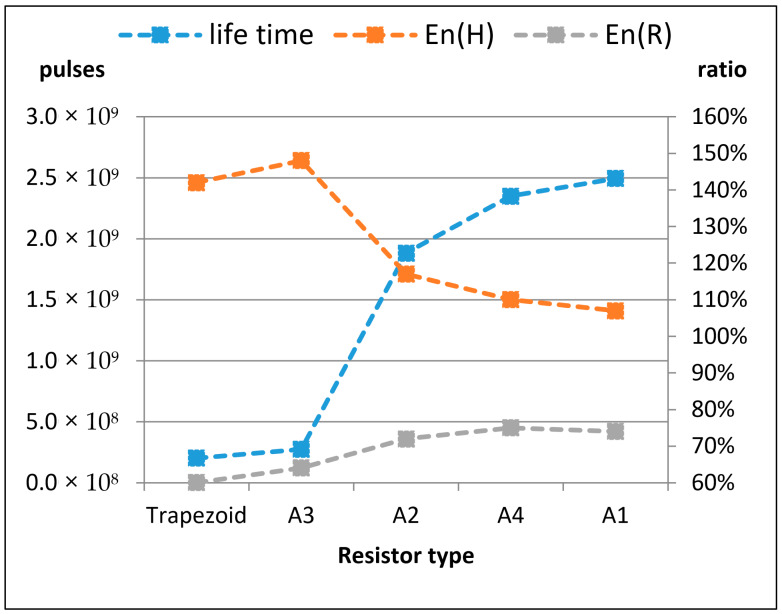
Experimental lifetime results and electric fields simulation results; the resistor shapes are shown in Figure 3.

The number of pulses were counted by a counter mechanism on the circuit, which ceases its operation when the resistor burns out, as can be seen in Figure 7. As explained and seen in Einat et al’s research [28], the burnout starts at the hotspot (point H in Figure 1), and continues rapidly by a thermal runaway process until the resistor is disconnected. The experiment results are shown in Table 2, which presents the different resistors and their respective average number of pulses until breakdown. The results in this table are arranged according to the number of pulses (noted as “lifetime”) and the simulated results are added again for the convenience of relating the experimental lifetime to the simulated electric fields. The results are also presented graphically in Figure 8.

In Figure 6. The following trends are seen. There is a ~15% degradation in the resistor–conductor interface normalized electric field *En*(*R*), which should optimally be as small as possible. But at the same time, the trend of the normalized electric field *En*(*H*) at the hot spot was improved by ~30%. As seen in the results, with these trends, the number of pulses until breakdown was increased by more than an order of magnitude, from 2 × 108 to 2.5 × 109. This is a major improvement obtained only by choosing the geometry of the resistor ramp properly. It is understood that the hot spot failure is more dominant. Improving the *En*(*H*) factor, even at the expense of the *En*(*R*), leads to an overall major improvement. 

## 5. Discussion and Conclusions

In this research, theoretical and experimental work was done to test the effect of the micro resistor geometry on its lifetime. A clear correlation between the geometry and lifetime of a MB resistor arises from the experimental results, with the differences in lifetime spanning an order of magnitude. These results are supported by a theoretical analysis which identified two failure mechanisms—the extreme field gradients near the ramp–body joining vertex (hot spot, *H*) and near the center of the conductor–ramp interface (*R*).

The experimental results show that the best predicting factor is the value of the normalized field at the hot spot *E_n_*(*H*). It can be seen that when *E_n_*(*H*)is made smaller, the resistor lifetime improves immensely—even when the normalized field at the conductor–ramp interface *E_n_*(*R*) is made greater as a result. This tradeoff is apparent in the designs shown in this research, but further modeling and experimentation may yield techniques to improve both normalized field values at once. 

The experimental study was done using 30 Ω sheet resistance of the resistive layer, but the same effect is expected regardless of the exact value of the sheet resistance, since it is depended on the geometry. 

These results have immediate ramifications regarding the different printing apparatuses which function with thermal inkjet technology. After development and refining, large plates of printer head arrays can be realized with sizes reaching that of any LCD screen. There would be an improvement in both the resolution and speed of the print [29], and the inherently longer MTBF would allow refilling to become practical. In addition, 3D printing apparatuses can greatly benefit from a similar scale-up as entire layers of ink could be printed simultaneously [30], thereby shortening printing time from hours to minutes. The improvement in resistor lifetime can create new opportunities where large-scale, refillable thermal inkjet apparatuses are economically viable.

## Figures and Tables

**Table 1:** Simulation results; the resistor shapes are shown in Figure 3.

Shape	*E*(*C*) (v/m)	*E*(*H*) (v/m)	*E*(*R*) (v/m)	*E_n_*(*H*)	*E_n_*(*R*)
A1	371.3	397	274.2	107%	74%
A2	377.3	443.3	273.2	117%	72%
A3	397.9	587.3	256.6	148%	64%
A4	368.4	404.7	278	110%	75%
Trapezoid	415.2	588.7	247.1	142%	60%

**Table 2:** Experiment results sorted by lifetime achieved in comparison to the simulated normalized electric fields.

Resistor Shape	*E_n_*(*R*)	*E_n_*(*H*)	Average Number of Pulses until Breakdown
Trapezoid	60%	142%	2.02 × 10 ^8^
A3	64%	148%	2.75 × 10 ^8^
A2	72%	117%	1.88 × 10 ^9^
A4	75%	110%	2.35 × 10 ^9^
A1	74%	107%	2.49 × 10 ^9^
